# Supplementary material for: Phasor field diffraction based reconstruction for fast non-line-of-sight imaging systems
Source: Nat Commun. 2020 Apr 2;11:1645. doi: 10.1038/s41467-020-15157-4 (PMC7118131; doi:10.1038/s41467-020-15157-4)
Supplement: Supplementary file 1 — Supplementary Information [file 41467_2020_15157_MOESM1_ESM.pdf]

Supplementary Information

Phasor Field Diffraction Based Reconstruction for Fast Non-Line-of-Sight Imaging Systems

Xiaochun Liu[1], Sebastian Bauer[2], Andreas Velten[1,2,*]

[1]*Department of Electrical and Computer Engineering, University of Wisconsin – Madison*
[2]*Department of Biostatistics and Medical Informatics, University of Wisconsin – Madison*
**Correspondence should be directed to velten@wisc.edu*

This supplemental document contains the following sections:

Supplementary Note 1: Additional results

Supplementary Note 2: Additional tables

Supplementary Note 3: Algorithm pseudocode

Supplementary Note 4: Additional SPAD array and confocal capture description

Supplementary Note 1

This section contains all additional results for the main paper.

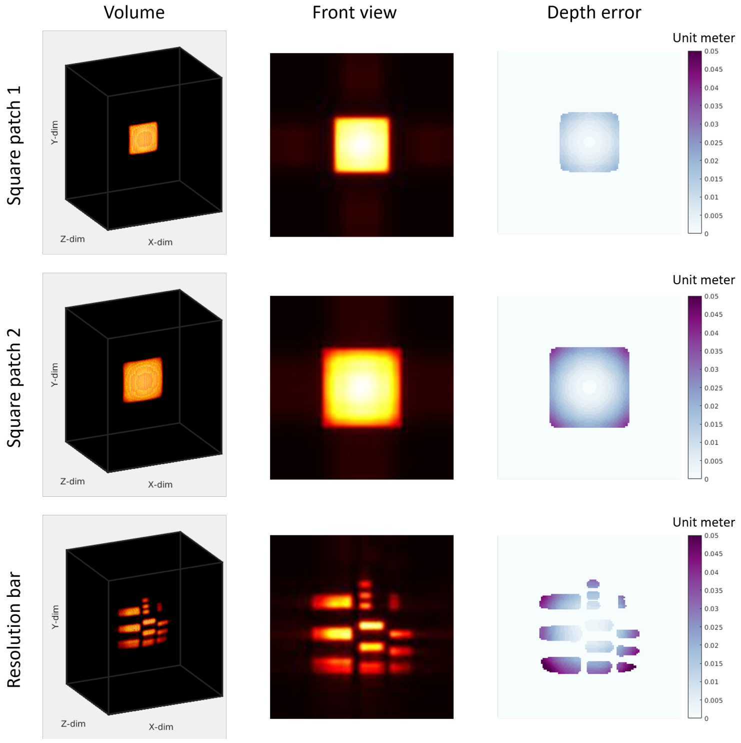

Supplementary Figure 1: Additional results from simulated non-confocal datasets [1]. Three simulated targets at 0.5 m distance from a 1 m by 1 m relay wall with a single SPAD position located at the center. For each target, we display results as a 3D volume, a 2D front view image by choosing the maximum intensity along the depth direction and the corresponding depth error in meters. From the front view image, a 2D irradiance map of the hidden target is reconstructed. The virtual camera exhibits distortions similar to the ones seen in real cameras. Since the resulting depth error is preserved for different scenes it can likely be calibrated if more accurate depth is desired. The error appears to be consistent across the different scenes with a variation of less than one voxel. The root-mean-square error values for three simulated targets are 0.0097 m, 0.0178 m and 0.0257 m, respectively.

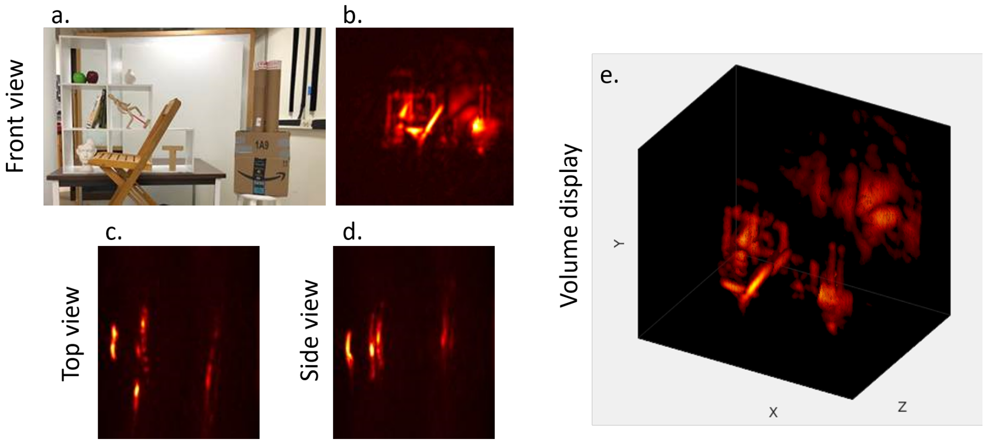

Supplementary Figure 2: An additional three dimensional volume rendering of the 20 ms office scene result used in the main paper. **a.** shows the captured geometry. **b-d.** show the reconstructed image by the maximum intensity projection from front, top and side views. **e.** shows three dimensional volume of the reconstruction.

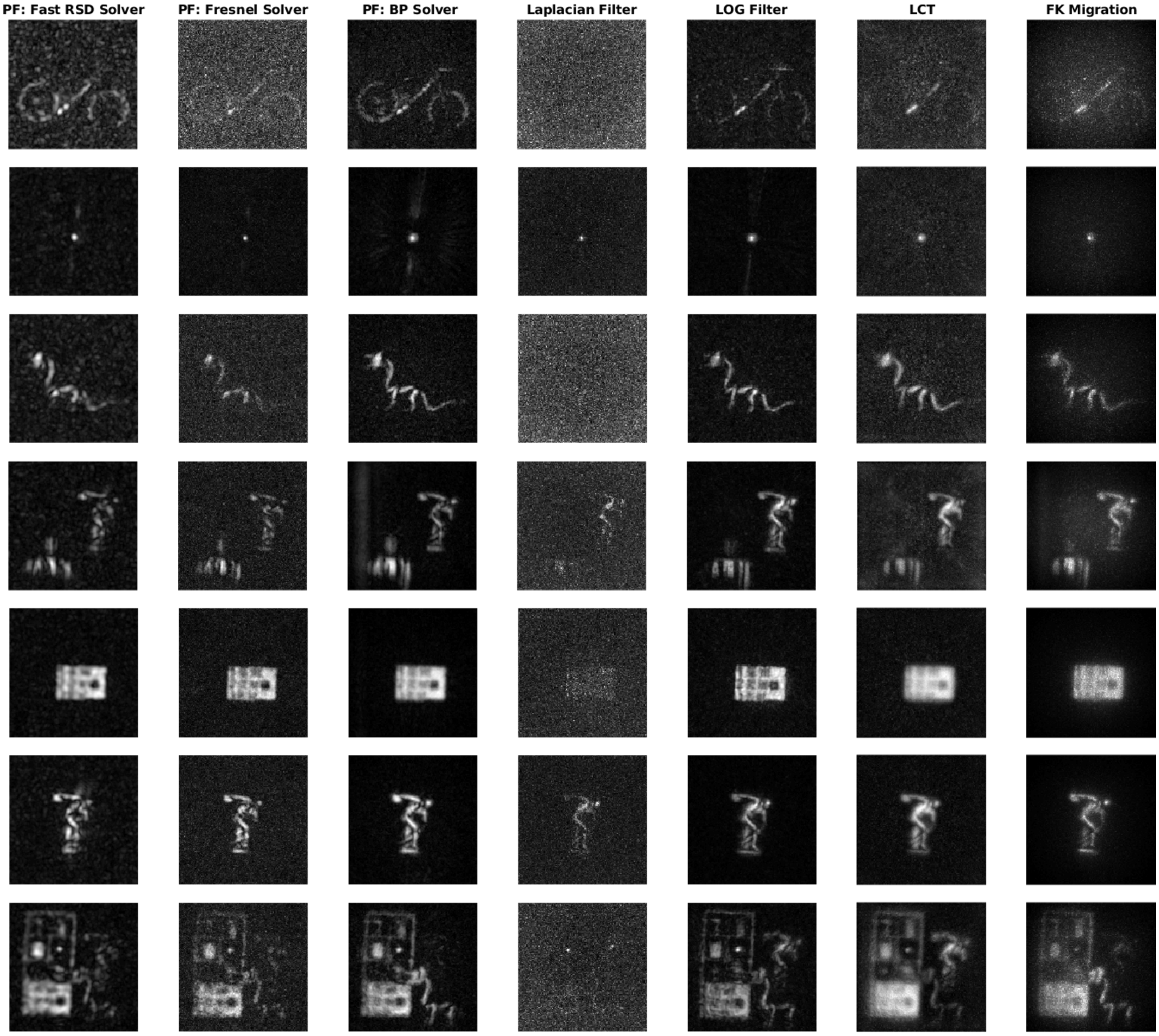

Supplementary Figure 3: Comparisons on confocal scanning shortest exposure datasets [2]. The first three columns correspond to the phasor field (PF) NLOS method [3] out of which the first two columns present our fast implementation (one with RSD, one with Fresnel diffraction kernel[3]) and the third column shows the results using the convolution backprojection kernel calculated from the LCT [4]. The fourth (Laplacian filter) and fifth (LOG: Laplacian of Gaussian) columns are filtered backprojection with filter implementation from [3] and the backprojection step is calculated from the convolution provided by LCT. The last two columns show LCT and FK-Migration [2]. For the shortest exposure dataset, we interestingly find out that LOG is quite robust. The Fresnel diffraction solver seems suited for confocal data, although it does not perform well on non-confocal data [3].

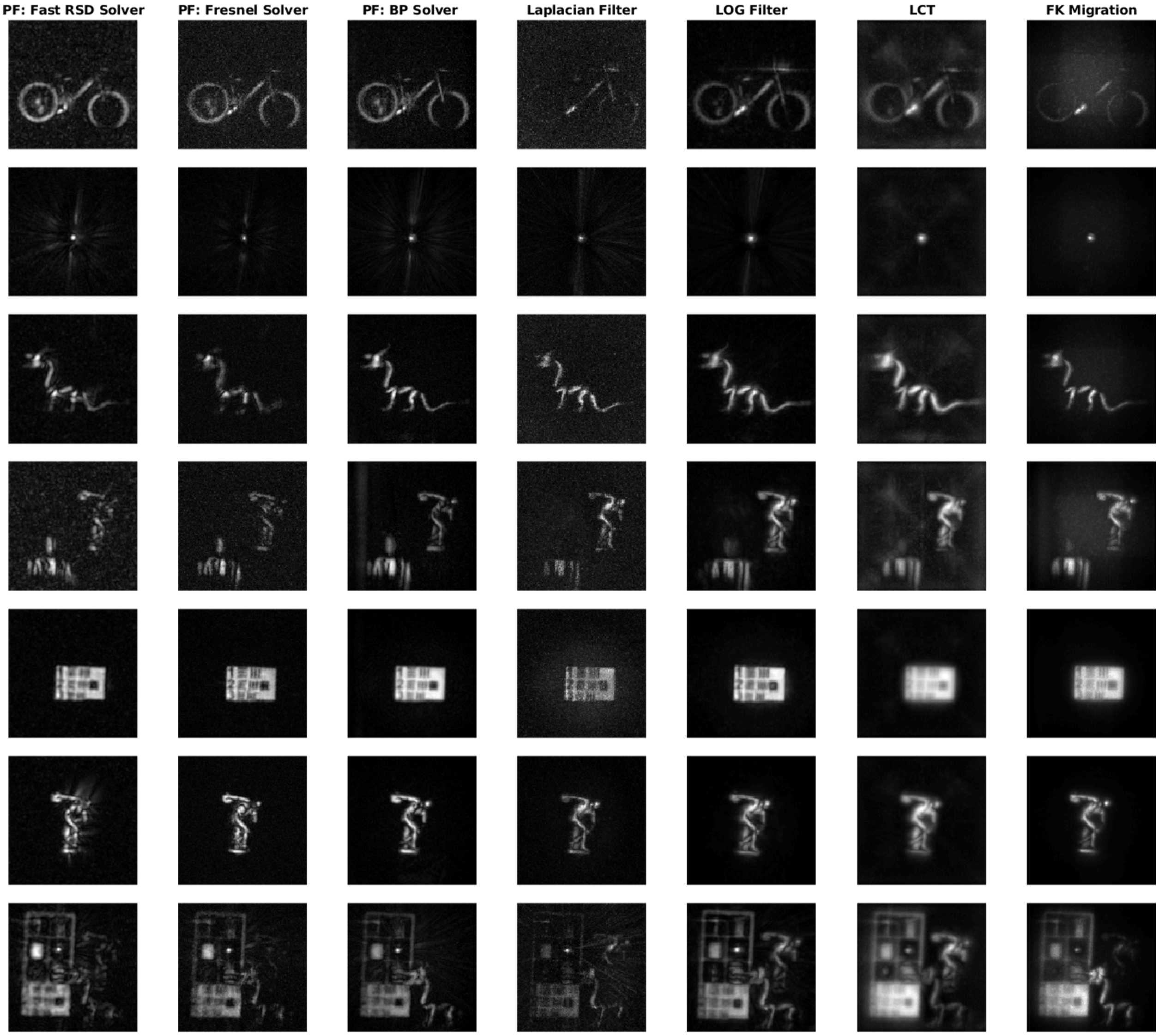

Supplementary Figure 4: Comparisons on confocal scanning longest exposure datasets [2]. The first three columns correspond to the phasor field (PF) NLOS method [3] out of which the first two columns present our fast implementation (one with RSD, one with Fresnel diffraction kernel [3]) and the third column shows the results using the convolution backprojection kernel calculated from the LCT [4]. The fourth (Laplacian filter) and fifth (LOG: Laplacian of Gaussian) columns are filtered backprojection with filter implementation from [3] and the backprojection step is calculated from the convolution provided by LCT. The last two columns show LCT and FK-Migration [2]. For the longest exposure dataset, almost all methods perform well. The Fresnel diffraction solver seems suited for confocal data, although it does not perform well on non-confocal data [3].

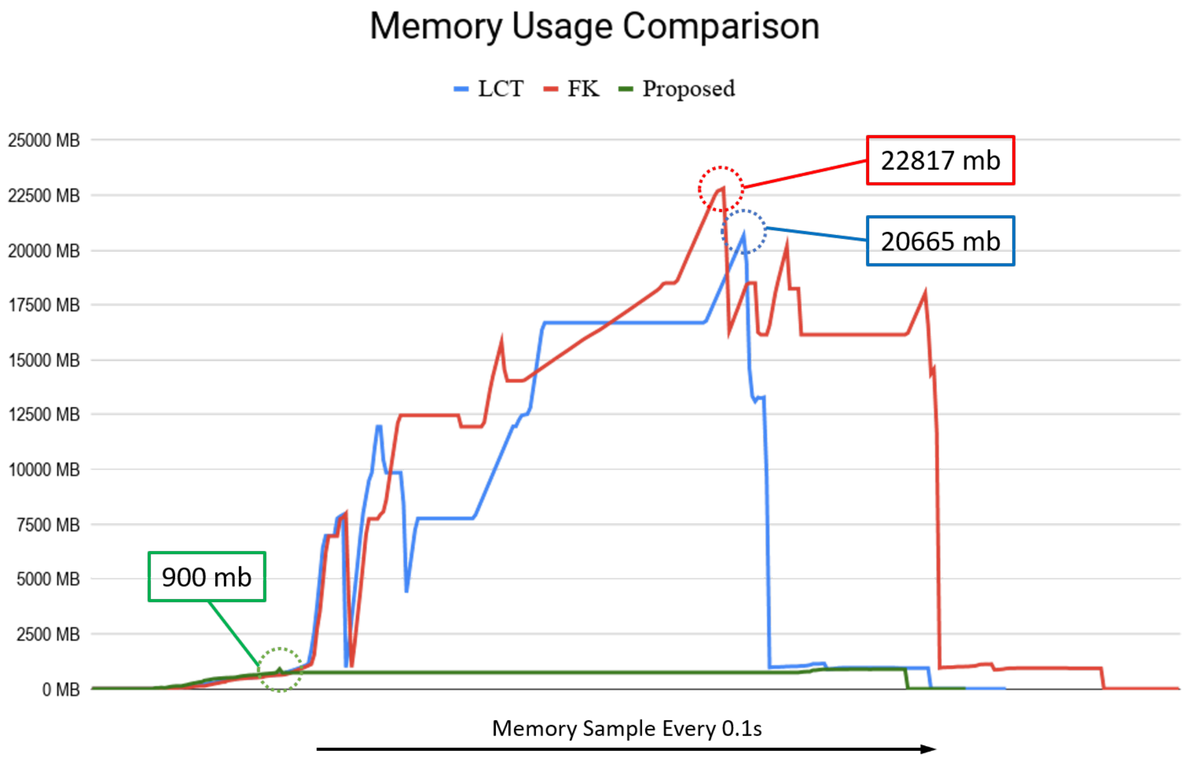

Supplementary Figure 5: Memory usage comparison. This plot shows the memory usage of our proposed method as well as LCT and FK Migration while they are running. We sample the used memory every 0.1 s and start acquisition 4 s before each method starts running. Notice that all un-optimized methods are running in MATLAB non-GUI mode on the same PC (32 GB memory) which requires 750 MB as baseline. We also mark the peak-memory point for each method.

Supplementary Note 2

This section contains all additional tables for the main paper.

Office Scene Acquisition Time	Proposed 1 section	Proposed 2 sections	Direct Integration	Approx. LCT (low res)	Approx. FK (low res)
1 ms	19.9 s	30.1 s	8248 s	15.6 s (3.80 s)	22.4 s (5.52 s)
5 ms	15.2 s	24.3 s	8685 s	15.7 s (3.76 s)	22.5 s (5.52 s)
10 ms	15.7 s	24.7 s	8534 s	15.8 s (3.80 s)	22.9 s (5.5 s)
20 ms	16 s	23.9 s	8667 s	15.5 s (3.79 s)	22.3 s (5.51 s)
1000 ms	18.7 s	37 s	5776 s	15.5 s (3.74 s)	22.4 s (5.5 s)

Supplementary Table 1: Office Scene run time comparison. This table shows the actual run time for generating the results in main paper Fig. 7. Our proposed method starts from the captured wavefront and has the same volume size as the Direct Integration method (150 x 150 x 125 voxels). For showing the best reconstruction quality of the approx LCT and approx FK methods, we use a voxel grid of 256 x 256 x 512 with 1 cm sampling resolution on the relay wall. Approx LCT and approx FK can be much faster when down-sampling the spatial dimensions as shown in brackets (128 x 128 x 512), but the results are even more blurry than the ones shown in main paper Fig.7. Note that down-sampling the spatial domain is not possible, as the number of spatial voxels has to equal the number of time bins and lower time resolution would lead to even worse results (but faster run time). The flexibility of adapting the full 3D voxel grid is an advantage of our RSD algorithm.

Dataset	Proposed	Direct Integration	Approx. LCT (low res)	Approx. FK (low res)
4	2.9 s	1298 s	15.5 s (3.7 s)	21.8 s (5.5 s)
44i	2.8 s	1316 s	15.4 s (3.72 s)	22.1 s (5.5 s)
NLOS	2.9 s	1292 s	15.6 s (3.69 s)	23 s (5.47 s)
Resolution Bar	2.9 s	1290 s	15.4 s (3.71 s)	25 s (5.49 s)
Shelf Light On	2.7 s	1302 s	15.3 s (3.67 s)	22.3 s (5.55 s)

Supplementary Table 2: Simple scenes run time comparison. This table shows the actual run time for generating the results in main paper Fig. 8. Our proposed method starts from the captured wavefront and has the same volume size as the Direct Integration method. For showing the best reconstruction quality of the approx LCT and approx FK methods, we use a voxel grid of 256 x 256 x 512 with 1 cm sampling resolution on the relay wall. Approx LCT and approx FK can be much faster when down-sampling the spatial dimensions as shown in brackets (128 x 128 x 512).

Dataset	Scene Depth	Material
Officescene<br>1 ms, 5 ms, 10 ms, 20 ms	0.5 m - 2.5 m	Wooden chair, white shelf, cardboard, books, plastic, white board, statue ...
Officescene<br>1000 ms	0.5 m - 2.5 m	Wooden chair, white shelf, cardboard, books, plastic, white board, statue ...
4	1 m	White styrofoam
44i	0.5 m - 1.3 m	White styrofoam
NLOS	0.75 m	White styrofoam
Resolution Bar	0.75 m	White styrofoam
Shelf Light On	0.8 m	White shelf, cardboard, books, plastic ...

Supplementary Table 3: Target scene parameters. This table contains: scene depth complexity (distance away from the relay wall), target materials.

Dataset	λ (cm)	β	Pulse Separation (m)	Number of Fourier components	Depth Min (m)	Depth Max (m)	Reconstructed Aperture Size (m)
Office Scene 1 ms	6	4	6	117	0.5	3	3
Office Scene 5 ms	6	5	6	91	0.5	3	3
Office Scene 10 ms	6	5	6	91	0.5	3	3
Office Scene 20 ms	6	5	6	91	0.5	3	3
Office Scene 1000 ms	4	5	6	139	0.5	2.5	3
4	4	5	3	69	0.5	1.5	2
44i	4	5	3	69	0.5	1.5	2
NLOS	4	5	3	69	0.5	1.5	2
Resolution Bar	4	5	3	69	0.5	1.5	2
Shelf, light on	4	5	3	69	0.5	1.5	2

Supplementary Table 4: Additional Parameters for reconstructions. This table contains the additional parameters used for each dataset to obtain the results shown. λ: phasor field virtual wavelength, β: number of modulation periods per Gaussian pulse. *Pulse separation*: spacing between periodic pulses. *Number of Fourier components*: number of discrete frequencies used in the reconstruction. *Depth Min, Depth Max* and *Reconstructed Aperture Size* describe the reconstruction volume. All results are obtained using $2\,\text{cm} \times 2\,\text{cm} \times 2\,\text{cm}$ voxels during reconstruction.

Supplementary Note 3

This section contains the algorithm pseudocode for the main paper. The discrete RSD propagator function is provided in Algorithm 1, the 4D moving wavefront using RSD in Algorithm 2, the spatial sectioning using RSD in Algorithm 3 and a memory efficient implementation in Algorithm 4.

Algorithm 1: Function for discrete RSD propagator

Parameter Description:
$u1$: input phasor field wavefront
sL: side length (physical length) for $u1$, unit m
λ: corresponding phasor field $u1$ wavelength, unit m
z: propagation distance, unit m
$u2$: output phasor field wavefront

Function *[$u2$]* = `propRSD(`$u1$, sL, λ, z`)`**:**

$[M, \text{-}] = \textit{size}(u1)$ `// get input field square matrix size`

`/* Spatial Sampling Interval */`
$dx = sL/(M-1)$ `// uniform horizontal and vertical sampling`

`/* Discretize depth */`
$\hat{z} = z/dx$
$\mu = \lambda \cdot \hat{z}/(M \cdot dx)$

`/* Center grid coordinate */`
$m = \textit{linspace}(0, M-1, M)$; $m = m - M/2$
$n = \textit{linspace}(0, M-1, N)$; $n = n - M/2$

`/* Define mesh grid */`
$[g_m, g_n] = \textit{meshgrid}(n,m)$

`/* Compute 2d RSD diffraction kernel */`
`/* Need to adjust` $j2\pi$ `to` $-j2\pi$ `based on sign for distance variable` z `*/`
$g = \exp(-j2\pi(\hat{z}^2 \cdot \textit{sqrt}(1 + (g_m^2/\hat{z}^2 + g_n^2/\hat{z}^2))/(\mu \cdot M)))/\textit{sqrt}(1 + g_m^2/\hat{z}^2 + g_n^2/\hat{z}^2)$

`/* Calculate the output field */`
$G = \textit{fft2}(g)$
$U1 = \textit{fft2}(u1)$
$U2 = U1.{*}G$ `// Fourier domain multiplication`
$u2 = \textit{ifftshift}(\textit{ifft2}(U2))$
return $u2$

Algorithm 2: 4D moving wavefront according to Eq. (10) in our main paper

Data: Input phasor field wavefront $u1$, output plane depth z_{v}, time shift vector $\mathbf{t}_{\mathrm{loop}}$

Result: Output phasor field wavefront $u2$ at plane z

/* $\mathbf{t}_{\mathrm{loop}}$ stands for the time shifted variable, 0 s defines the illumination position */

for t *in* $\mathbf{t}_{loop}$ **do**
  for ω *in* $\mathbf{\Omega}$ **do**
    u2 += $e^{j\omega t} \cdot \mathcal{R}_z[u1, z]$; // $\mathcal{R}_z$ stands for RSD function based on specific implementation
  end
end

Algorithm 3: Spatial sectioning according to Eq. (14) in our main paper

Data: Input phasor field wavefront $u1$, output plane depth z, illumination phasor field temporal window length D, light source position $\mathbf{x}_{ls}$, spatial section number L, speed of light c

Result: Output phasor field wavefront $u2$ at plane z

Parameter Description:;
$\mathbf{x}$: stands for 3x1 vector in Euclidean coordinate system to represent the spatial position;

/* Calculate Error Map $E(\mathbf{x})$ */

for x_{tmp} *in* $\mathbf{x}$ **do**
  $E(x)$ = *norm*(x_{tmp} - $\mathbf{x}_{ls}$) - z;
end

/* Assign spatial mask $M(\mathbf{x})$ and travel time B based on spatial section number L */

for i=*1 to* L **do**
  $B_i = (L-1)D$;
  $M(\mathbf{x}, i) = (E(\mathbf{x}) > (i - \frac{3}{2})D)$.*($E(\mathbf{x}) \leq (i - \frac{1}{2})D$)
end

for ω *in* $\mathbf{\Omega}$ **do**
  for $i = 1$ *to* L **do**
    $u2$ += $M(\mathbf{x}, i) \cdot e^{j\frac{\omega}{c}(z+B_i)} \cdot \mathcal{R}_{\mathbf{x}_{\mathrm{v}}}[u1]$; // $\mathcal{R}_{\mathbf{x}_{\mathrm{v}}}$ stands for RSD function based on specific implementation
    ;
  end
end

Algorithm 4: Memory Efficient Implementation for 2D Image Recovery

Data: Input phasor field wavefront $u1$, output shifted depth slice $\mathbf{d}_{\text{loop}}$

Result: Output phasor field 2D image $I(\mathbf{x})$

```
initialization;
I(x) = zeros(x) ;            // Initialize zeros for the final image
/* d_loop refers to the reconstructed volume size in depth */
for d in d_loop do
    u_tmp(x) = zeros(x) ;    // Temporal variable for each depth slice
    for ω in Ω do
        /* Sectioning method described above (or proper
           inverse diffraction step) */
        Wavefront calculation for u_tmp(x) using u1 at depth d;
    end
    /* Choose maximum intensity value for each spatial pixel
       in the output image */
    I(x) = max(abs(u_tmp(x)), I(x))
end
```

Supplementary Note 4

In this section we review the fundamental constraints on NLOS capture and provide an outlook for future NLOS SPAD array sensors. This section is intended to motivate the development of the non-confocal NLOS reconstruction algorithm that is the main contribution of this paper. The section is not in itself intended as a contribution and experimental demonstration of the signal behavior described here is subject to future work.

Recent reconstruction methods compute reconstructions from data using single pixel gated SPADs. This is a choice of necessity as gated SPAD arrays were not available until very recently. Among those methods are confocal scans of the relay wall, i.e. scans where the laser illumination is directed to one patch on the relay surface and light is collected only from the same patch. Single pixel and confocal scanning are common in applications like LiDAR and confocal microscopy where a maximum signal for the first bounce return is desired and multibounce and ambient light signals need to be suppressed. In these applications all returning first light comes from the illuminated surface and point scanning comes with no penalty in detection efficiency. On the contrary, concentrating all available light onto one pixel helps to reduce interference from multibounce light and elevate the collected signal over the noise floor.

However, an NLOS imaging measurement is very different as light returns simultaneously from the entire surface of the relay wall. A single pixel detector with high spatial resolution collects light only from a very small fraction of the relay surface at a time. Even if all the illumination light is directed towards one point on the relay surface, returning third bounce light still arrives from everywhere on the relay wall, see Supplementary Figure 6. An NLOS algorithm that can only utilize light returned from the small area that was illuminated therefore can only utilize a vanishingly small fraction of the 3rd bounce photons available for capture. In this respect, NLOS imaging is similar to passive ambient light imaging methods such as conventional cameras. In these systems light arrives simultaneously from the entire field of view. A point scanning photography camera would be vastly inferior to a focal plane array and therefore is rarely used. Similarly, single pixel scanning NLOS measurements have severe disadvantages over methods using array sensors to the point that they are unlikely to be of practical use as soon as capable array sensors are available.

The fundamental problem is thus that there is a limited finite number of photons per area reflecting off the relay wall. For a given aperture size, the maximum possible photon rate achieved in the measurement is thus inversely proportional to the area of relay wall used. Since the largest area a single pixel transient can be collected from without blurring is limited to about 1 cm^2, one can only increase the area and thereby the photon rate by using multiple pixels collecting multiple transients simultaneously. This is entirely independent of particular technical implementations of the sensor and optics and their nuances and represents a fundamental physical constraint.

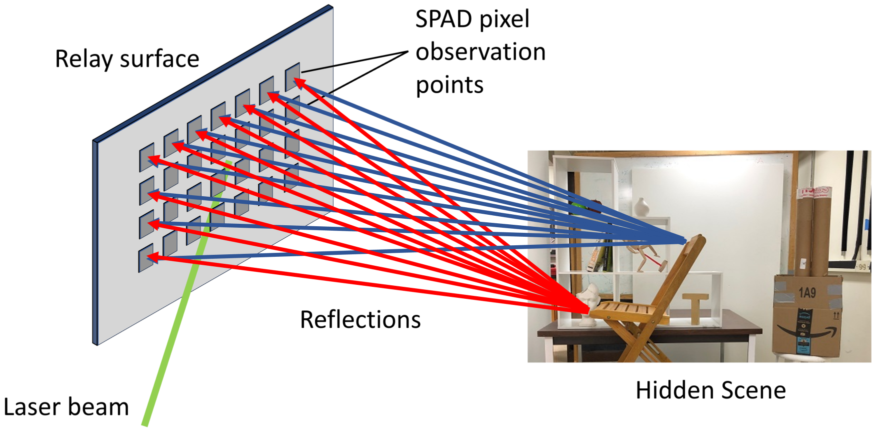

Supplementary Figure 6: Scene objects reflect light to every location on the relay wall. Non-confocal acquisition of NLOS imaging measurements with SPAD array detectors allows for the simultaneous capturing of light reflected off all such relay wall locations.

Next let us now consider the engineering limitations of actual lenses and SPAD sensors. For optimal light collection we would like each SPAD pixels to collect light from an area of $< 1\,\mathrm{cm}$ diameter on the relay wall. Larger areas would blur the collected time responses while smaller areas would unnecessarily reduce the amount of light collected by the pixel.

First generation commercial SPAD array sensors (Princeton Lightwave, MPD SPC3) are limited by choices made in their manufacturing and by the available technology at the time of their design. Due to the necessity to fit all processing electronics and memory associated with SPAD detection on a 2D sensor chip around each pixel, the fill factors and light sensitive pixel areas of these early SPAD arrays are extremely small. This is not actually a problem when the relay surface is large and far away ($> 100\,\mathrm{m}$), as a lens with adequate magnification can be chosen such that each pixel observes a 1 cm diameter patch as desired. Detected patches on the relay wall would be sparse, but this does not represent a substantial problem for NLOS reconstructions. When the imaging system is positioned close to the relay wall, however, the required focal length of the objective to achieve the required magnification would be unrealistically small, so in lab experiments, a sensor with a much larger pixel area is required. This is why the single pixel SPAD with a pixel diameter of 50 micron is better suited for lab experiments at close stand-off distances.

Active area sizes for these first generation arrays are chosen to detect sufficient light in typical long distance LiDAR applications while minimizing the total sensor area and thereby the cost. The rest of the array design is subject to similar trade-offs. Because the arrays are designed for LiDAR applications they are optimized to detect the first arriving photon for a continuous uniform array of pixels with a reasonable time resolution (200 ps is already more than enough to outperform most other LiDARs). In a 2D chip, only very simple electronics for detection and storage of a photon event can be used to not further decrease the fill factor. The total rate of photon timings that can be

read of the device are determined by the choice of readout interface.

Since the design of this first generation of SPAD arrays, 3D stacking methods have become widely available. It is now possible to place some of the SPAD electronics in separate layers behind the pixels. Second generation arrays provide 256 by 256 or 512 by 512 pixels with a fill factor of around 10%. To collect sufficient amounts of photons for real time reconstructions we anticipate needing only about 100 pixels. For optimal operation we require a custom designed SPAD array with lower spatial resolution and lower number of pixels, but better time resolution and a slightly higher total count rate. There are numerous possible designs that can achieve these numbers. Higher time resolutions similar to the ones of the current single pixel SPAD can be achieved by keeping crucial components off chip, for example in an FPGA or multi channel ASIC. Since the required count rate per pixel is relatively low, counting electronics can be shared by multiple pixels.

The first benefit of using a SPAD array sensor rather than a single pixel for 3rd bounce imaging is thus similar to the benefit of using a lens over a pinhole. While it does not provide any additional information, a lens collects orders of magnitude more light. In current systems with relay wall sizes of up to 2 m by 2 m, a single pixel that can only collect light from 1 cm^2 at a time only detects 1/40,000 of the available signals. However, capturing with an array provides additional information. Rather than just enabling virtual cameras, it allows us to design virtual camera projector pairs that can analyze light from the location of the relay wall and separate components of 4 or more bounces. We will consider this scarcely explored area in future work.

Supplementary References

1. M. Galindo, J. Marco, M. O'Toole, G. Wetzstein, D. Gutierrez, and A. Jarabo. A dataset for benchmarking time-resolved non-line-of-sight imaging. In *International Conference on Computational Photography (ICCP)*. IEEE, 2019. Dataset link: https://graphics.unizar.es/nlos.

2. D. B. Lindell, G. Wetzstein, and M. O'Toole. Wave-based non-line-of-sight imaging using fast f-k migration. *ACM Trans. Graph. (SIGGRAPH)*, 38(4):116, 2019.

3. X. Liu, I. Guillén, M. La Manna, J. H. Nam, S. A. Reza, T. H. Le, A. Jarabo, D. Gutierrez, and A. Velten. Non-line-of-sight imaging using phasor-field virtual wave optics. *Nature*, 572(7771):620–623, 2019.

4. M. O'Toole, D. B. Lindell, and G. Wetzstein. Confocal non-line-of-sight imaging based on the light-cone transform. *Nature*, 555(7696):338, 2018.
